# Flow Cytometry of Human Primary Epidermal and Follicular Keratinocytes

**Published:** 2008-02-19

**Authors:** Alfredo Gragnani, Michelle Zampieri Ipolito, Christiane S Sobral, Milena Karina Coló Brunialti, Reinaldo Salomão, Lydia Masako Ferreira

**Affiliations:** Laboratory of Cell Culture, Division of Plastic Surgery, Federal University of São Paulo (UNIFESP), São Paulo, Brazil; Laboratory of Immunology of Division of Infectious Diseases, Federal University of São Paulo (UNIFESP), São Paulo, Brazil

## Abstract

**Objective:** The aim of this study was to characterize using flow cytometry cultured human primary keratinocytes isolated from the epidermis and hair follicles by different methods. **Methods:** Human keratinocytes derived from discarded fragments of total skin and scalp hair follicles from patients who underwent plastic surgery in the Plastic Surgery Division at UNIFESP were used. The epidermal keratinocytes were isolated by using 3 different methods: the standard method, upon exposure to trypsin for 30 minutes; the second, by treatment with dispase for 18 hours and with trypsin for 10 minutes; and the third, by treatment with dispase for 18 hours and with trypsin for 30 minutes. Follicular keratinocytes were isolated using the standard method. **Results:** On comparing the group treated with dispase for 18 hours and with trypsin for 10 minutes with the group treated with dispase for 18 hours and with trypsin for 30 minutes, it was observed that the first group presented the largest number of viable cells, the smallest number of cells in late apoptosis and necrosis with statistical significance, and no difference in apoptosis. When we compared the group treated with dispase for 18 hours and with trypsin for 10 minutes with the group treated with trypsin, the first group presented the largest number of viable cells, the smallest number of cells in apoptosis with statistical significance, and no difference in late apoptosis and necrosis. When we compared the results of the group treated with dispase for 18 hours and with trypsin for 10 minutes with the results for follical isolation, there was a statistical difference in apoptosis and viable cells. **Conclusion:** The isolation method of treatment with dispase for 18 hours and with trypsin for 10 minutes produced the largest number of viable cells and the smallest number of cells in apoptosis/necrosis.

The epidermis is formed by multiple layers of keratinocytes and its differentiation process begins in the proliferative basal layer. Keratinocytes are the main cell type of the epidermis, and they are responsible for the formation of the cutaneous barrier.

There are 3 populations of keratinocytes present in the epidermis: stem cells, transit amplifying cells, and differentiated cells.^1^ Proliferative cells produced in the basal layer are responsible for the renewal of this tissue. These are formed by a small population of stem cells and also by transit amplifying cells.^2^

In the stratum corneum, keratin and glycolipids are arranged in successive layers forming the cutaneous barrier, an impermeable functional unit that maintains life. The loss of liquids, proteins, and electrolytes through lesions, as a consequence of the absence of this barrier, leads to important fluid loss, causing hemodynamic instability and also allowing invasion by several pathogenic microorganisms. Depending on how extensive is the cutaneous loss, as in severe burn injuries, this mechanism plays an important role in the morbidity and mortality of these patients.^3^

Diseases that lead to a major skin loss, such as extensive degloving injuries, cutaneous aplasia, epidermolysis bullosa, giant congenital nevi, pyoderma gangrenosum, traumas, and congenital alterations, are ideal for permanent wound coverage with cultured keratinocytes when there is a lack of sufficient skin graft donor sites for wound closure. In burn wounds, large skin surfaces are frequently affected, leaving no donor site available.^4^, ^5^

The main objective of the treatment of the burn patient is recovery of the lost cutaneous coverage. Major burn patients hardly survive for too long without the cutaneous coverage because of a high infection rate, as the wound contraction and reepithelialization take a long time to be completed. In major burns, donor sites are scarce. Therefore, permanent wound coverage is done with cultured keratinocytes, which allows the patients to survive.

The use of a single layer of cultured keratinocytes onto the wound has the disadvantage of presenting with a low graft integration rate and the resulting long immobilization period to allow for wound healing. After the end of this process, new lesions may occur because of the graft fragility.^6^

In the traditional protocol used for isolation of keratinocytes, the efficiency rate of colony formation is very low, about 3% to 4%, producing a very small number of proliferative cells from an initial skin fragment removed from the major burn patient.^7^–^9^

These proliferative cells are part of the transit amplifying cell population that belongs to an intermediate stage between the epidermal stem cells and the differentiated cells. This proliferative cell population is found in the epidermis, from which a sample was taken for primary isolation and culture of keratinocytes. A defined population of proliferative cells is also found in the hair follicles, which is the main site for proliferation of keratinocytes for coverage of partial thickness skin injuries.

A keratinocyte isolation method that would lead to the formation of a higher number of viable proliferative cells is essential to the success of culture techniques and major burn treatment, since it would produce large amounts of tissue for permanent wound coverage in a short time.

The use of enzymes, such as trypsin and dispase among others, associated with the mechanical factor in the isolation of cells from a skin fragment is essential for the culture method. However, it is also necessary that the technique yields viable cells and not dead or apoptotic cells.

To better understand the development of the culture of human keratinocytes, it is necessary to characterize the different features of this cell. Since one of the objectives in a cell culture laboratory is improving the culture method, the use of a flow cytometer is of fundamental importance in studying the cellular dynamics of keratinocytes.

For many years, biochemical and biophysical measurements of isolated cells have been taken basically by visual analysis with the use of several types of microscope. Many of the methods used for immunohistochemical and cellular analysis became more and more sophisticated after the development of flow cytometers. Flow cytometry is a modern technique used for cellular analysis and it enables simultaneous measurements of multiple parameters, providing a large amount of cellular information from a single sample.

Among the examples of measurements that can be taken, physical characteristics, such as size, volume, and refraction and viscosity indexes, as well as chemical characteristics, such as the contents of DNA, RNA, proteins, and enzymes, and antigenic characteristics, such as cell surface molecules that are easily detected by immunofluoroscence using monoclonal antibodies, should be emphasized.^10^

Flow cytometry also allows for a better characterization of cells, making possible the study of the nucleus, its membrane, the cell cycle, cellular apoptosis, and necrosis using specific markers.^11^ Other advantages of flow cytometry are the objectivity and reproducibility of results and cell-specific analysis of a larger number of cells, being a technique that is independent of the operator since the parameters are calibrated by the instrument.

The aim of this study was to characterize cultured human primary keratinocytes isolated from epidermis and hair follicles by different methods.

## METHODS

### Culture of human keratinocytes

The primary culture of keratinocytes started with a suspension of isolated cells derived from a full thickness skin of a patient. The suspension was cut into smaller pieces and treated with trypsin, dispase, or a combination of dispase and trypsin to dissociate the cells.

The keratinocyte culture medium was a 3:1 mixture of 750 mL Dulbecco's Modified Eagle's Medium (DMEM) (high glucose [4.5 g/L], L-glutamine [584 mg/L], and sodium pyruvate [110 mg/L]) and 250 mL Ham's F-12 medium (Sigma-Aldrich, St. Louís, Mo), totaling a volume of 1 L. This was supplemented with 10% fetal bovine serum (FBS) (Invitrogen, Grand Island, NY) and 24 mg of freshly prepared adenine (6-aminopurine hydrochloride) diluted in 20 mL DMEM/Ham's F-12 mixture, resulting in a final concentration of 1.8 × 10^−4^ mol/L. One millilitre of 10^−10^ mol/L cholera toxin (*Vibrio cholerae*, Type Inaba 569 B), 2 mL of penicillin/streptomycin (100 UI/mL, 100 μ g/mL) (Sigma), 2 mL of 0.4 μ g/mL hydrocortisone, 1 mL of 2 × 10^−9^ mol/L transferrin/triiodo-L-thyronine (Sigma-Aldrich), and 1.3 mL of 5 μ g/mL insulin (porcine) in a final concentration of 5 μ g/mL. The pH was adjusted to approximately 7.2; the medium was sterilized with a 0.22-μ m filter, kept in a refrigerator at 4°C, and produced a red color.

Normal human keratinocytes were isolated from the skin of patients who underwent surgical procedures in the Plastic Surgery Division at the Federal University of São Paulo (UNIFESP/EPM). The 1-cm^2^ fragments of total skin discarded in the surgical center were obtained in the morning when the cell isolation was performed. The skin fragments were obtained only after the patients were informed about the purpose for which the skin was collected, agreed, and signed an informed consent form. The study was approved by the Ethics Committee of UNIFESP/EPM.

Three different techniques of isolation of epidermal keratinocytes were applied to 3 groups of skin fragments. The traditional technique, applied to the first group, has been described by Green et al^7^ and Green^8^, ^9^ and was implemented at the Laboratory of Cell Culture of the Plastic Surgery Division at the UNIFESP/EPM by Gragnani et al.^12^, ^13^

The first step in a laminar flow hood was to wash the skin fragments in tubes containing phosphate buffered saline (PBS) (Sigma) and then dissociate them in 0.05% trypsin (Sigma-Aldrich). The smaller fragments were placed in a preheated shake flask containing 6 mL of trypsin and 6 mL of versene (Gibco BRL, New York), totaling a volume of 12 mL. After 30 minutes under mechanical agitation and enzyme action at 37°C, the isolation of keratinocytes was accomplished.

The supernatant was removed from the solution containing the isolated cells, the solution was centrifuged to remove trypsin and Versene, and the cells were resuspended in 3 mL of keratinocyte culture medium.

In the technique of isolation using dispase (Roche Diagnostics Corporation, Indianápolis, Ind), applied to the second group of skin fragments, each fragment was subjected to the same washing procedure as was used in the first group, using 30 mL of PBS in eight 50-mL conical tubes to remove contaminants. Following washing, the fragments were placed in a conical tube containing 15 mL of 0.5% dispase for 18 hours at 4°C. Then, 5 mL of Versene was added, the tube content was transferred to a petri dish, and the epidermis was separated from the dermis using 2 delicate clamps. The epidermis was placed in a tube containing 5 mL of 0.25% trypsin for 5 minutes at 37°C. Then, it was blended in a Vortex mixer for 30 seconds, placed again in an incubator for 5 minutes at 37°C, and homogenized by repeated pipetting 30 times. Finally, decantation of the supernatant and centrifugation were performed as in the previous group.

In the technique of isolation applied to the third group, dispase was used in a similar way as in the second group. After separating the epidermis from the dermis, the epidermal fragments were placed in a petri dish containing 0.05% trypsin and the isolation was performed for 30 minutes as in the first technique.

The protocol for isolation of follicular keratinocytes started with the removal of the follicles from the scalp, discarded from rhytidoplasty, with 2 delicate clamps and movements perpendicular to the surface of the skin fragments to expose the follicle. The follicular material was directly collected in a 60-mm culture dish containing 5 mL of keratinocyte culture medium. The distal region of the follicle was cut with iris scissors and only the bulge and bulb regions of the hair follicles were collected.

Following this, all hair follicles were placed in another 60-mm culture dish. For each 50~follicles, 1 mL of 0.05% trypsin was added. The dish was kept in an incubator for 8 minutes at 37°C. Then, the dish was examined under an inverted microscope and the dissociation of keratinocytes from the follicular bulb was observed. Next, the remaining follicles were removed and immediately 1 mL of keratinocyte culture medium was added for each 1 mL of trypsin in order to neutralize the enzyme action.^14^

The set consisting of keratinocytes, trypsin, and culture medium was placed in a 15-mL tube and centrifuged at 800 rpm for 6 minutes. After centrifugation, the cells were resuspended in 1 mL of PBS and analyzed in a flow cytometer.

### Flow cytometry

The flow cytometry technique was used to detect the cell cycle and apoptosis in suspension cells, using propidium iodide (PI) (BD Biosciences, San Jose, Calif) and Annexin V (BD Biosciences), as in the method described by Nicoletti et al.^15^ The method involved staining the cellular DNA with PI, using 500 μL of buffer solution (10 mmol/L Hepes/NaOH, pH 7.4, 140 mmol/L NaCl, and 2.5 mmol/L CaCl_2_) (Sigma) added to 10 μL of 50 μg/mL PI buffer solution (BD Biosciences).

Cells were resuspended in 100 μL of buffer solution containing PI, incubated for 10 minutes in the dark at room temperature, washed with the buffer solution, and immediately analyzed in the flow cytometer.

To use the Annexin V marker, 2 × 10^6^ cells/mL resuspended in PBS were centrifuged after the isolation of keratinocytes. The cell pellet was resuspended in 1 mL of Annexin V buffer solution diluted 10-fold in distilled water. Tubes were prepared according to the cell population assayed and cells were resuspended in Annexin V buffer solution. In the first tube was placed 100 μL of cell suspension with 5 μL of Annexin V and 10 μL of PI; in the second tube, 5 μL of Annexin V; in the third tube, 10 μL of PI; and in the fourth tube, only the cell suspension without markers. The solutions were blended in a Vortex mixer and incubated for 20 minutes in the dark at room temperature. The results from tubes 1 and 4 were observed and analyzed. The experiments using flow cytometry were performed in triplicate.

The statistical analysis of flow cytometry was done in the Cell Quest program, which gives us the results in percentage of cells. One-way analysis of variance (ANOVA) was carried out to test the comparison between age groups, using Bartlett's test for equal variances and Tukey's multiple comparison test. Two-way ANOVA was carried out to study the viability of cells, using Bonferroni posttests.

## RESULTS

**Figure 1:**
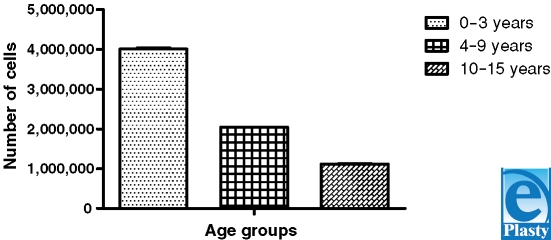
Analysis of age groups and the total number of cells after primary isolation of keratinocytes.

In order to define the flow cytometry protocol, primary keratinocytes isolated from skin fragments of 22 patients aged between 0 and 15 years and divided in 3 age groups, ranging from 0 to 3 years, 4 to 9 years, and 10 to 15 years, were evaluated. It was observed that the largest number of isolated cells, approximately 4 × 10^6^ cells, were obtained from the age group 0 to 3 years and that the other age groups showed a decrease in the number of isolated cells inversely proportional to the age of the patients, that is, the older the patients in a given group, the lower the number of isolated cells obtained from that group (Fig 1). The results were significant when we compared the 0–3 years group with the other two groups and the 4–9 years group with the 10–15 years group (Table 1).

**Figure 2:**
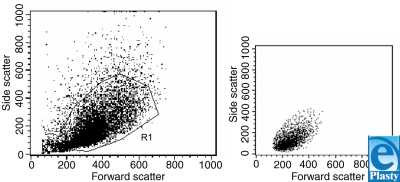
Forward scatter (cell size) vs side scatter (cell complexity) dot plot generated by a flow cytometer, showing the cell distribution of human cultured primary keratinocytes (R1), which was looked up in the right section of the figure.

The flow cytometer allowed the measurement of parameters that are represented graphically as a correlation between forward scatter (cell size) and side scatter (cell complexity), showing regions of higher cellular frequency corresponding to specific populations studied (Fig 2).

**Figure 3:**
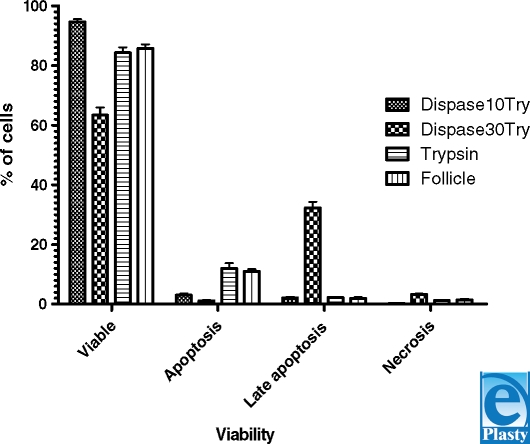
Types of primary isolation of epidermal keratinocytes and the percentage of viable cells, apoptosis, late apoptosis, and necrosis.

After the definition of the flow cytometry protocol for primary keratinocytes, the experiment was performed applying the 3 methods for primary isolation of keratinocytes using different sequences of enzymes and the method for isolation of keratinocytes from follicles. The results of cell viability in each method studied are presented in Figure 3. When we compared the group treated with dispase (18 hours) and trypsin (10 minutes) with that treated with dispase (18 hours) and trypsin (30 minutes), the first group presented the largest number of viable cells, the smallest number of cells in late apoptosis and necrosis with statistical significance, and no difference in apoptosis. When we compared the group treated with dispase (18 hours) and trypsin (10 minutes) with the group treated with trypsin, the first group presented the largest number of viable cells, the smallest number of cells in apoptosis with statistical significance, and no difference in late apoptosis and necrosis. When we compared the results of the group treated with dispase (18 hours) and trypsin (10~minutes) with the results of follicle isolation, the first group presented the largest number of viable cells, the smallest number of cells in apoptosis with statistical significance, and no difference in late apoptosis and necrosis (Table 2).

## DISCUSSION

Since the 1970s, with the publication of the work by Rheinwald and Green,^16^ serial culture of human keratinocytes became possible. Since then, several authors have presented different techniques for isolation of keratinocytes in order to optimize the established culture protocols.^17^–^19^

Different enzymes were used in cell isolation procedures in order to improve the efficiency of the primary culture of keratinocytes.^20^ The final objective of these studies was bringing about an improvement in the primary culture protocols.^21^

Recently, new studies have been performed to find out whether there is a link between the isolation of keratinocytes and the age of patients.^22^–^24^ Lack of such a link (keratinocytes' proliferation rate with ageing) is clear in the literature, so the choice of age groups in this study was not to study this fact. Significant results to evaluate improvements in keratinocytes' isolation should be true to all age groups because the behavior of cells related to the proliferation rate is similar in every age to the trauma that they suffer from isolation and it is directly related to the toxicity of enzymes used in the process, which results in more or less cell death.

In this age group, 0–15 years, we can also find facilities to have skin fragments from foreskins, which give us almost the same size of fragments, a 1 cm^2^ of skin, with a similar average weight. These differences were not important to the results because before the flow cytometry evaluation, we counted the number of cells and a fixed number of cells, 2 × 10^6^cells/mL, were submitted to cytometry.

The 3 age groups used in this study were related to the fact that 50% of the burns occur in the 0–18 years age group, resulting in a high death percentage. In this experiment, it was found that the number of isolated viable cells decreased as the age of the patients increased, with statistical significance, which agrees with the findings in the literature.^23^, ^24^

Traditionally, the number of live cells in a suspension is estimated by counting, microscopically, the cells that exclude an acidic dye, such as trypan blue. The method can be adapted for the flow cytometer by using PI, which is excluded by viable cells and, when taken up by dead or dying cells, binds to nucleic acids and fluoresces red. The use of flow cytometry has the advantage that a large number of cells can be counted quickly and that the determination of negative/positive cells is objective.

Apoptosis induces a variety of changes in the plasma cell membrane, including changes in permeability and alterations in the membrane lipids. During apoptosis, there is a major change in the membrane lipids in that phosphatidyl serine (PS) residues flip from the internal to external membrane. PS binds Annexin V and these changes can be observed by incubating unfixed cells with Annexin V.

Annexin V is a protein that has a high affinity for negatively charged phospholipids, such as PS, in the presence of Ca^2+^ ions. It is customary to add PI to distinguish cells that have lost integrity of the plasma membrane.

So, if the cells are negative to PI and Annexin V, they represent viable cells. If they are negative to PI and positive to Annexin V, they are cells in apoptotic state. If they are positive to PI and Annexin V, they are in late apoptotic state, because Annexin V express the PS residues but the DNA is exposed and they also express the PI. And when they are positive to PI and negative to Annexin V, they are in necrotic state, because PS is not present to bind Annexin V.^25^

Different procedures for isolation of keratinocytes were evaluated in this study. The cell group treated with trypsin for 30 minutes followed standard protocol.^7^–^9^, ^12^, ^13^ The cell groups treated with dispase were also subjected to trypsin treatment. Dispase was used since it separates the epidermis from the dermis on the basement membrane zone by cleaving the protein junctions between desmosomes and dermal anchoring fibrils. In this way, keratinocytes of the basal layer, where the majority of cells responsible for epidermal proliferation are found, become more exposed to the action of trypsin. This fact is also true when thermolysin is used, so both dispase and thermolysin can expose the basal layer and the proliferative keratinocytes to the action of trypsin, which needs less time to separate these proliferative cells in the isolation process, diminishing the toxicity of trypsin and cell death.^26^

In the analysis by flow cytometry, when comparing results of cell treatments with dispase-trypsin (10 minutes) and dispase-trypsin (30 minutes), it was observed that the dispase-trypsin (10 minutes) group presented the largest number of viable cells, the smallest number of cells in late apoptosis and necrosis with statistical significance, and no difference in apoptosis.

When comparing isolation of epidermal keratinocytes results obtained with dispase-trypsin (10 minutes) and with the standard method, it was observed that the dispase-trypsin (10 minutes) method presented the largest number of viable cells and the smallest number of cells in apoptosis. The set of analysis for late apoptosis and necrosis showed no statistical difference.

Since both the traditional method and the dispase-trypsin (30 minutes) method produced worse results than the dispase-trypsin (10 minutes) method, there was no need to compare them. The method that involved cell treatment with dispase for 18 hours and with trypsin for 10 minutes was pointed as the most efficient in producing viable cells and less damage, such as cells in necrosis and late apoptosis.

Therefore, the results obtained with the method that involved treatment with dispase-trypsin (10 minutes) were chosen to be compared with the results obtained from the traditional method applied to follicular keratinocytes. The sets of analysis for apoptosis and viable cells provided statistically significant results.

These experiments were used as a first approach in trying to characterize which method of cell isolation would produce the largest number of viable cells and indirectly the largest number of stem cells.^27^

The experiments were done in triplicate, and the remanescent cells, which were not used in the flow cytometry or were cryopreserved, were seeded on flasks to evaluate the behavior after passages. It was observed that the dispase (18 hours) and trypsin (10 minutes) group gave a significantly higher number of colonies in the flasks until fourth passage after rhodamine staining.

An extension of this study, with the use of specific markers for stem cells,^28^, ^29^ could yield precise data that would allow for changes in the protocol for keratinocyte isolation, making possible the isolation of adult stem cells by flow cytometry for use in tissue engineering.

## Figures and Tables

**Table 1:** Statistical analysis of the total number of cells within age groups

One-way analysis of variance			
*P* value	<.0001		
*P* value summary	^*^		
Are means significantly different? (*P* < .05)	Yes		
Number of groups	3		
*F*	2719		
*R*^2^	0.9965		
Bartlett's test for equal variances			
Bartlett's statistic (corrected)	8.133		
*P* value	0.0171		
*P* value summary	†		
Do the variances differ significantly? (*P*0 < .05)	Yes		
Tukey's multiple comparison test	Mean difference	*q*	Significant? *P* < .05?
0–3 y vs 4–9 y	1,965,000	69,000	Yes
0–3 y vs 10–15 y	2,886,000	101,4	Yes
4–9 y vs 10–15 y	921,400	31,33	Yes

**Table 2:** Statistical analysis of 4 types of primary isolation of epidermal keratinocytes and the percentage of viable cells, apoptosis, late apoptosis, and necrosis

Source of variation	% of total variation	*P* value	*P* value summary	Significant?
*Two-way analysis of variance*				
Interaction	6,95	<.0001	^*^	Yes
Mean	0,00	.09994	NS	No
SD	92,97	<.0001	^*^	Yes
*Bonferroni posttests*				
Dispase10try vs dispase30try				
**SD**	**Dispase10try**	**Dispase30try**	**Difference**	**95% CI of difference**
Viable	94,67	63,33	−31,33	−34.27 to −28.40
Apoptosis	3,000	1,100	−1,900	−4.838 to 1.038
Late apoptosis	2,133	32,23	−30,10	27.16 to 33.04
Necrosis	0,2000	3,333	3,133	0.1955 to 6.071
**SD**	**Difference**	**t**	***P* value**	**Summary**
Viable	−31,33	32,91	<.001	^*^
Apoptosis	−1,900	1,996	>.05	NS
Late apoptosis	30,10	31,62	<.001	^*^
Necrosis	3,133	3,291	<.01	†
Dispase10try vs trypsin				
**SD**	**Dispase10try**	**Trypsin**	**Difference**	95% CI of difference
Viable	94,67	84,33	−10,33	−13.27 to −7.395
Apoptosis	3,000	12,00	9,000	6.062 to 11.94
Late apoptosis	2,133	2,233	0,1000	−2.838 to 3.038
Necrosis	0,2000	1,233	1,033	−1.905 to 3.971
**SD**	**Difference**	***t***	***P* value**	**Summary**
Viable	−10,33	10,85	<.001	^*^
Apoptosis	9,000	9,454	<.001	^*^
Late apoptosis	0,1000	0,1050	>.05	NS
Necrosis	1,033	1,085	>.05	NS
Dispase10try vs follicle				
**SD**	**Dispase10try**	**Follicle**	**Difference**	95% CI of difference
Viable	94,67	85,67	−9,000	−11.94 to −6.062
Apoptosis	3,000	10,97	7,967	5.029 to 10.90
Late apoptosis	2,133	1,900	−0,2333	−3.171 to 2.705
Necrosis	0,2000	1,467	1,267	−1.671 to 4.205
**SD**	**Difference**	***t***	***P* value**	**Summary**
Viable	−9,000	9,454	<.001	^*^
Apoptosis	7,967	8,368	<.001	^*^
Late apoptosis	−0,2333	0,2451	−.05	NS
Necrosis	1,267	1,331	>.05	NS

Dispase10try indicates dispase (18 hours), trypsin (10 minutes); dispase30try, dispase (18 hours), trypsin (30 minutes).
